# ﻿Plume moths (Lepidoptera, Pterophoridae) of a recently discovered lepidopteran diversity hotspot in the Mount Cameroon area, with descriptions of four new species

**DOI:** 10.3897/zookeys.1231.139530

**Published:** 2025-03-11

**Authors:** Peter Ustjuzhanin, Vasily N. Kovtunovich, Sylvain Delabye, Vincent Maicher, Szabolcs Sáfián, Eric B. Fokam, Robert Tropek

**Affiliations:** 1 Altai State University, Lenina 61, Barnaul, RU-656049, Russia Altai State University Barnaul Russia; 2 Biological Institute, Tomsk State University, Lenina Prospekt 36, Tomsk 634050, Russia Tomsk State University Tomsk Russia; 3 University of the Free State (UFS), 205 Nelson Mandela Dr., Park West, Bloemfontein, Free State, South Africa University of the Free State (UFS) Bloemfontein South Africa; 4 Department of Ecology, Faculty of Science, Charles University, Viničná 7, CZ-12843 Prague, Czech Republic Institute of Entomology, Biology Centre of the Czech Academy of Sciences České Budějovice Czech Republic; 5 Institute of Entomology, Biology Centre of the Czech Academy of Sciences, Branišovská 31, CZ-37005 České Budějovice, Czech Republic Charles University Prague Czech Republic; 6 The Nature Conservancy Gabon, Impasse Edowangani, Libreville, Gabon The Nature Conservancy Gabon Libreville Gabon; 7 Hungarian Natural History Museum, Department of Zoology, Baross utca 13, H-1088 Budapest, Hungary Hungarian Natural History Museum, Department of Zoology Budapest Hungary; 8 Department of Animal Biology and Conservation, Faculty of Science, University of Buea, P.O. Box 63, Buea, Cameroon University of Buea Buea Cameroon

**Keywords:** Afrotropics, biodiversity, Cameroon, microlepidoptera, new species, Pterophoridae, taxonomy, tropical rainforest

## Abstract

Moth diversity on Mount Cameroon, a critical biodiversity hotspot in the Afrotropics, remains understudied despite the region’s rich and unique ecosystems. In this study, 34 species of plume moths (Pterophoridae) were recorded from the Mount Cameroon region, including four species new to science: *Titanoptilusbigoti* Ustjuzhanin & Kovtunovich, **sp. nov.**, *Titanoptilusmurkwe* Ustjuzhanin & Kovtunovich, **sp. nov.**, *Hellinsiaekonjo* Ustjuzhanin & Kovtunovich, **sp. nov.**, and *Hellinsiamapanja* Ustjuzhanin & Kovtunovich, **sp. nov.** Images of the adult type specimen and male genitalia for *Titanoptilusmelanodonta* Hampson, 1905 are published for the first time. In addition, 25 species are reported as new records for the Cameroonian fauna, raising the number of known Pterophoridae species in the country from 19 to 48. The results significantly expand our understanding of plume moth diversity in the region and extend the known distribution range of several species. These findings emphasise the importance of Mount Cameroon as a biodiversity hotspot within the Afrotropics. Enhanced conservation efforts are essential to preserve the unique biodiversity of Mount Cameroon, especially considering threats such as ongoing habitat degradation in some parts of the region and climate change.

## ﻿Introduction

Mount Cameroon (4,040 m a.s.l.), an active volcano in southwestern Cameroon, is recognised as one of the key centres of insect diversity within the Afrotropics, particularly for lepidopterans (Ustjuzhanin et al. 2018, 2020a, 2024; Larsen 2005). Its position within the biodiversity-rich Guineo-Congolian forest zone, combined with its gradients in elevation (ranging from sea level to 4,040 metres) and precipitation (from its southwestern foothills, which rank among the three rainiest places in the world, to relatively dry savannahs in rain shadows on its northeastern slopes; Cable and Cheek 1998; Maicher et al. 2020a), supports highly heterogeneous habitats that accommodate large numbers of both widespread and endemic species of insects and other taxa (e.g., Ustjuzhanin et al. 2018; Delabye et al. 2020). Moreover, due to its inaccessibility and, later, its legal protection as part of the Mount Cameroon National Park, large portions of Mount Cameroon’s ecosystems have remained relatively well-preserved from the negative effects of human activity (Cable and Cheek 1998).

Several lepidopteran groups, including butterflies (e.g., Sáfián and Tropek 2016; Maicher et al. 2018, 2020a, b; Delabye et al. 2021), various microlepidopterans (Ustjuzhanin et al. 2018, 2020a, 2024), and other moth groups (e.g., Maicher et al. 2016, 2020a, b; Yakovlev and Sáfián 2016; Przybyłowicz et al. 2019a, b; Delabye et al. 2020; Mertens et al. 2021), have been the focus of recent studies on Mount Cameroon, revealing the exceptional diversity of these taxa on the mountain. Notably, prior research has identified Mount Cameroon as a hotspot for many-plumed moths (Alucitidae), significantly advancing our understanding of their Afrotropical biodiversity (Ustjuzhanin et al. 2018, 2020a, 2024). Despite this progress, many lepidopteran groups remain understudied in the Mount Cameroon region, highlighting the need for further exploration of its insect diversity.

Pterophoridae, commonly referred to as plume moths, represent one such understudied family in the region. Characterised by their often deeply cleft wings, which give them their distinctive “plume-like” appearance, this group has a global distribution but remains insufficiently documented in the Afrotropics (De Prins and De Prins 2025). Knowledge of plume moths in Cameroon is still very limited, as no comprehensive studies on this family have been conducted in the country. To date, only 19 species have been recorded from Cameroon (De Prins and De Prins 2025). By summarising plume moth material sampled over several years of ecological studies of moth communities in the Mount Cameroon region, including descriptions of new species and numerous new country records, this study provides the first comprehensive survey of Pterophoridae on Mount Cameroon.

## ﻿Materials and methods

We sampled plume moths on Mount Cameroon using standardised light trapping methods over multiple field seasons between 2014 and 2018, at localities spanning elevations from 30 to 2,400 m a.s.l. across the mountain’s southwestern, southern, and northern slopes. Light traps equipped with white and ultraviolet (UV) emitting bulbs were operated from dusk until dawn to attract moths. Specimens were collected manually from white sheets, euthanised using ammonium vapour, pinned, and preliminarily spread directly in the field for easier later processing, and stored dried with silica gel for transportation. The collected moths were identified based on external morphology and genitalia dissections; dissection and preparation of genitalia followed the standard lepidopteran taxonomic protocols described in Ustjuzhanin et al. (2018). The genitalia were mounted on permanent microscopic slides for further study, with unique identification codes assigned to each specimen for reference in taxonomic work. Holotypes will be deposited in the Nature Education Centre, Jagiellonian University, Kraków, Poland (**NECJU**), while paratypes and other specimens will be split between NECJU and the personal collections of P. Ustjuzhanin and V. Kovtunovich, located in Novosibirsk and Moscow, Russia (**CUK**). Morphological terminology follows Zagulajev (1986) and the distribution of individual species follows the Afromoths database (De Prins and De Prins 2025), unless specified otherwise.

The sampling localities are listed below in an alphabetical order:

- Bamboo Camp, 350 m a.s.l., Mount Cameroon (SW slope), 4.0879°N, 9.0505°E; a lowland rainforest with historical disturbances from selective logging.
- Bimbia-Bonadikombo, 30 m a.s.l., Bimbia-Bonadikombo Community Forest, Mexico Camp, 3.9818°N, 9.2625°E; littoral forest in the part of the community forest that is officially disturbance-free, but with intensive ongoing illegal logging (Ferenc et al. 2018).
- Crater Lake, 1,500 m a.s.l., Mount Cameroon (SW slope), 4.1443°N, 9.0717°E; submontane rainforest with a sparse canopy layer as a consequence of local disturbances by forest elephants (Maicher et al 2020b).
- Drink Gari, 650 m a.s.l., Mount Cameroon (SW slope), Drink Gari camp (also known as “Drinking Gari”), 4.1014°N, 9.0610°E; lowland rainforest with a dense canopy layer.
- Ekonjo, 1,500 m a.s.l., Mount Cameroon (S slope), 4.0881°N, 9.1168°E; upland closed-canopy rainforest belonging to the Ekonjo village.
- Ekonjo farmland, 800 m a.s.l., Mount Cameroon (S slope), 4.0687°N, 9.1311°E; farmlands and secondary growths surrounding the Ekonjo village.
- Elephant Camp, 1,850 m a.s.l., Mount Cameroon (SW slope), 4.1170°N, 9.0729°E; montane rainforest with a sparse canopy layer as a consequence of local disturbances by forest elephants (Maicher et al 2020b).
- Mann’s Spring, 2,200 m a.s.l., Mount Cameroon (SW slope), 4.1428°N, 9.1225°E; montane forest at the natural timberline.
- Mapanja, Mapanja camp, 1,850 m a.s.l., Mount Cameroon (S slope), 4.1157°N, 9.1315°E; montane forest with a mostly closed canopy layer, supplemented by small openings after fallen trees on a steep slope.
- PlanteCam, 1,100 m a.s.l., Mount Cameroon (SW slope), PlanteCam camp (also misspelled as “PlantiCamp” or “PlanteCamp”), 4.1175°N, 9.0709°E; upland rainforest in the transition between lowland and montane zones, with a sparse canopy layer as a consequence of local disturbances by forest elephants (Maicher et al 2020b).
- P&T antenna station, an area surrounding an antenna station of the Ministry of Post and Communications (P&T; sometimes misspelled as “PNT” by foreigners), 2,400 m a.s.l., Mount Cameroon (N slope), 4.21556°N, 9.2725°E; transition between montane forests and subalpine grasslands.

## ﻿Results

Altogether, we recorded 34 plume moth species in the Mount Cameroon area, four of which are described here as new to science. Additionally, we report 25 species (marked with *) as new records for Cameroon, as only five of the recorded species had been previously known from the country.

### ﻿Descriptions of the new species

#### ﻿ Titanoptilus bigoti

Taxon classificationAnimaliaLepidopteraPterophoridae

Ustjuzhanin & Kovtunovich sp. nov.

40468D87-49DE-5729-9F5E-8DCCFF831B9A

https://zoobank.org/8EA59C5A-694E-49B9-9B85-2004189371BE

Figs 1 , 2

##### Type material examined.

***Holotype*** • ♂ (NECJU No. 241015), Cameroon, Bamboo Camp, 350 m a.s.l., Mount Cameroon, 4.0879°N, 9.0505°E, 14–23.II.2016, Sz. Sáfián, R. Tropek, V. Maicher leg.

**Figures 1, 2.**
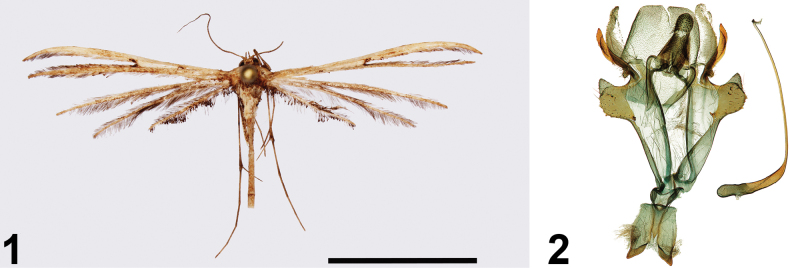
*Titanoptilusbigoti* Ustjuzhanin & Kovtunovich, sp. nov. **1** adult male, holotype, NECJU**2** male genitalia, holotype, NEJCU, preparation slide no. 241015. Scale bar: 10 mm.

##### External characters.

The wingspan is 31 mm. The head and tegulae are pale brown. The thorax is interspersed with dark-brown filiform scales. The labial palpi are thin, straight, and equal in length to the eye diameter, basally brown, and noticeably paler apically. The antennae are thin and dark brown. The forewings are pale brown, with a small elongated brown discal spot at the base. There is a distinct brown spot at the cleft base. The fringe inside the cleft is yellowish brown. The hindwings are yellowish brown. The hindwing fringes are dark, with the third lobe interspersed with evenly distributed dark spatulate scales on the costal margin and forming three distinct patches on the anal margin, a small cluster at the tip, two triangular patches along the middle, and a cluster at the base. The abdomen is long and pale brown, with creamy scales at the base of the thorax extending to the first abdominal segment. The hind legs are long and pale brown with noticeable darkening at the bases of the spurs.

##### Description.

***Male genitalia*.** The genitalia are symmetrical. The valves each comprise two lobes: the basal lobes arise beyond the sacculus are relatively wide and subquadrate, and narrow apically. The distal lobes are narrower, and longer, and ~ 2 × the length of the basal lobes. Narrow sclerotized processes extend from the distal lobes and are only slightly shorter than those lobes. The uncus is robust, equal in length to the distal lobes of the valves, basally wide, and apically narrow and rounded. The saccus is wide and triangular. Sternum VIII is forked, with sclerotised distal margins. The aedeagus is long and thin in its distal 2/3, bent at a right angle ~ 1/3 along its length. The basal part is noticeably thicker up to the bend.

##### Diagnosis.

**Figures 3, 4.**
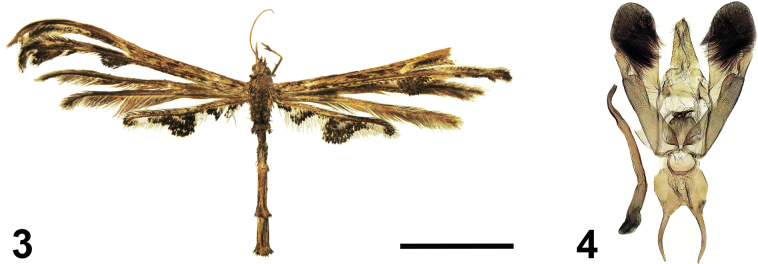
*Titanoptilusmelanodonta* Hampson, 1905 **3** adult male, holotype, NHMUK, locality: [Kenya], British East Africa, N’dimy, Uganda Railway, mile 469, leg. C. S. Betton **4** male genitalia, holotype, NHMUK, gen.pr. no.21811. Scale bar: 10 mm.

In terms of size and wings colour, the new species is similar to *Titanoptilusmelanodonta* Hampson, 1905 (Figs 3, 4) but differs in the absence of fringe bundles along the lower margin of the second lobe of the forewing and at the base of the third lobe of the hindwing. In the male genitalia, the new species is similar to *Titanoptilusprocerus* Bigot, 1969 (male genitalia described and illustrated in Bigot 1969), in having bilobed valves and the narrow, long, and bent aedeagus. However, in the new species, the valves are basally wide, and the aedeagus is bent at a right angle, while in *T.procerus*, the valves are basally narrow, and the aedeagus is arched.

##### Distribution.

Cameroon, Democratic Republic of Congo.

##### Flight period.

On Mount Cameroon, the species was collected in February.

##### Etymology.

The species is named in honour of Louis Bigot, the first researcher who worked on African plume moths, as a token of gratitude for his significant contributions.

##### Note.

In Bigot (1969), this species was misidentified and illustrated as *T.melanodonta* (collected in the Democratic Republic of Congo, d’Eala et de Bokuma, Equateur, leg. L. Bigot), because L. Bigot did not have an opportunity to study the *T.melanodonta* type specimen, and the species’ genitalia were not known at that time. Upon examining the male genitalia of *T.melanodonta* type specimen, collected in Kenya (Kenya, British East Africa, N’dimy, Uganda Railway, mile 469, leg. C.S. Betton; Figs 3, 4) and stored in the Natural History Museum in London, UK (**NHMUK**), PU and VK found that the male image in Bigot (1969) does not correspond to the type specimen. Therefore, we have illustrated the *T.melanodonta* holotype in this study (Figs 3, 4). Instead, the male genitalia image in Bigot (1969) exactly matches the newly described species. Although we do not have access to the specimens, we expect they belong to *T.bigoti*, described in this study, based on Bigot’s illustrations.

#### ﻿ Titanoptilus murkwe

Taxon classificationAnimaliaLepidopteraPterophoridae

Ustjuzhanin & Kovtunovich sp. nov.

EFA21366-D4A9-5026-ACF7-05825C5B32C2

https://zoobank.org/ACF340B5-2310-4B02-8D4D-321652E6AFE9

Figs 5 , 6

##### Type material examined.

***Holotype*** • ♀ (NECJU no. 241016), Cameroon, Crater Lake, 1,500 m a.s.l., Mt. Cameroon, 4.1443°N, 9.0717°E, 23–29.IV.2017, V. Maicher, P. Potocký, S. Delabye leg.

##### External characters.

**Figures 5, 6.**
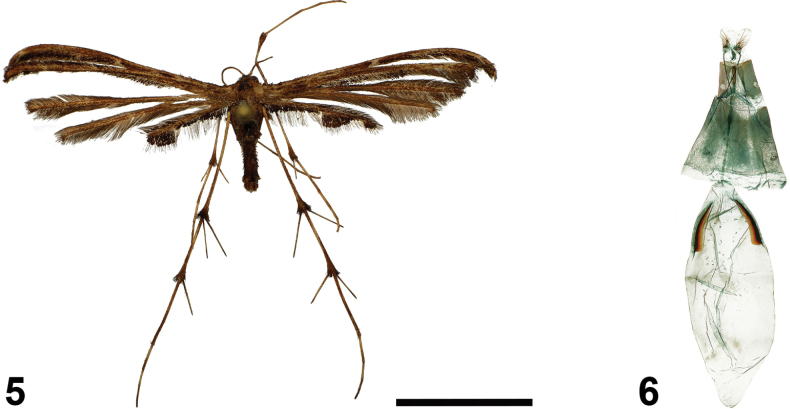
*Titanoptilusmurkwe* Ustjuzhanin & Kovtunovich, sp. nov. **5** adult female, holotype, NECJU**6** female genitalia, holotype, NEJCU, preparation slide no. 241016. Scale bar: 10 mm.

The wingspan is 36 mm. The head and thorax are dark brown, while the tegulae are pale brown. The labial palpi are thin, straight, and apically acute, with a length equal to the eye diameter. The antennae are thin and dark brown. The forewings are brown, with a narrow oblique yellowish stripe across the base of the first lobe. The first lobe is distally lightened with white scales, while the second lobe is mottled with brown and pale scales. The fringe inside the cleft is dark brown. Along the outer margin of the second lobe, the fringe is narrow and brownish grey but it widens and becomes darker in its distal part. The hindwings are brown, slightly paler than the forewings (unfortunately, not clearly visible in the specimen picture; Fig. 5). The fringe of the third lobe anal margin features a row of short dark spatulate scales on the basal half. These scales narrow further towards the middle before lengthening into a long, rounded scale bundle, which narrows again towards the apical fringe bundle, forming a rounded scale tooth at the lobe terminus. The hind legs are long and pale brown, with noticeable darkening at the bases of the spurs.

##### Description. *Female genitalia*.

The papillae anales are elongated and oval. The antrum is narrow, tubular, and sclerotized, with the ostium slightly extended. The ductus is thin, long, and membranous. The bursa copulatrix is long and oval, 2 × as long as the ductus, and contains paired robust sclerotized signa in its distal portion, each ~0.24 × the length of the bursa.

##### Differential diagnosis.

In size and wing colour, the new species is similar to *T.melanodonta*, but it differs in the absence of the wide portion of dark spatulate scales along the fringe of the outer margin under the cleft on the second lobe and the narrow basal row of spatulate scales among the fringes of the third lobe. In *T.melanodonta*, a wide scale tooth within the fringe of the anal margin is present under the forewing cleft, and the basal fringe of the third lobe of the hindwing forms a wide triangular scale tooth. In the female genitalia, the presence of signa in the bursa copulatrix distinguishes *T.murkwe* from other large species of the genus.

##### Distribution.

The species is only known from Cameroon.

##### Flight period.

The species was collected in April.

##### Etymology.

The species name is a noun in apposition, given in honour of the young and talented Cameroonian entomologist, Mercy Murkwe, who accompanied us during sampling of most specimens reported in this study.

#### ﻿ Hellinsia ekonjo

Taxon classificationAnimaliaLepidopteraPterophoridae

Ustjuzhanin & Kovtunovich sp. nov.

3A9691DE-3FDD-575A-BC8E-8BFD12098B2A

https://zoobank.org/65DE0D2E-884E-46CE-84DE-83E609AC0572

Figs 7–9

##### Type material examined.

***Holotype*** • ♂ (NECJU no. 241017), Cameroon, PlanteCam, 1,100 m a.s.l., Mount Cameroon, 4.1175000°N, 9.0709440°E, 09–14.IV.2015, V. Maicher, Sz. Sáfián, S. Janeček, R. Tropek leg.; ***Paratypes*** • 1♂, Cameroon, Buea, Mount Cameroon (SE slope), 09.XI.1986, G. Bassi leg. • 1♀, Cameroon, Crater Lake, 21.XI.2016, V. Maicher, Sz. Sáfián, S. Janeček, R. Tropek leg.; 3♀, 17–18.II.2017, P. Potocký, Sz. Sáfián, R. Tropek, J. Mertens, S. Janeček leg.; 1♂, 2♀, 23–29.IV.2017, V. Maicher, P. Potocký, S. Delabye leg. • 1♂, 1♀, Cameroon, Ekonjo, 24.X.2017, V. Maicher, S. Delabye leg. • 2♀, Cameroon, Elephant Camp, 19–24.XI.2014, V. Maicher, Sz. Sáfián, S. Janeček, R. Tropek leg.; 4♂, 2♀, 17–22.II.2017, P. Potocký, Sz. Sáfián, R. Tropek, J. Mertens, S. Janeček leg.; 1♀, 18–26.IV.2017, V. Maicher, P. Potocký, S. Delabye leg. •; 1♀, Cameroon, Mapanja, 14.V.2017, V. Maicher, P. Potocký, S. Delabye leg.; 2♂, 1♀, 23–28.X.2017, V. Maicher, S. Delabye leg.; 1♀, 23.X.2017, V. Maicher, S. Delabye leg. • 3♀, same data as the holotype; 1♂, 2♀, Cameroon, PlanteCam, 11–18.XII.2014, V. Maicher, Sz. Sáfián, S. Janeček, R. Tropek leg.; 1♂, 3♀, 29.I.–07.II.2016, Sz. Sáfián, R. Tropek, V. Maicher leg. (CUK, NECJU).

**Figures 7–9.**
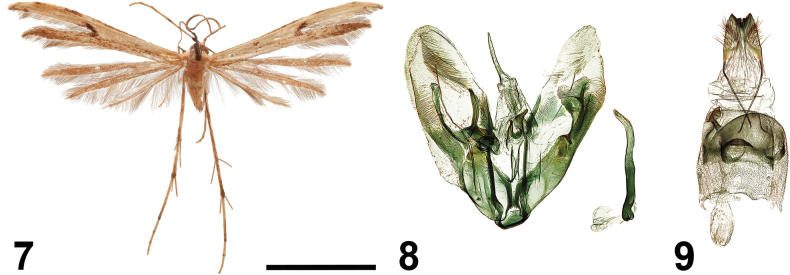
*Hellinsiaekonjo* Ustjuzhanin & Kovtunovich, sp. nov. **7** adult male, holotype, NECJU**8** male genitalia, holotype, NEJCU, preparation slide no. 241017 **9** female genitalia, paratype. Scale bar: 5 mm.

##### External characters.

The wingspan ranges from 15 to 19 mm, with the holotype measuring 17 mm. The thorax and tegulae are yellow. The head is covered with clinging yellow scales between the antennae. The collar is brown. The labial palpi are straight, short, and half the length of the eye diameter. The antennae are yellowish grey. The forewings are greyish brown, with the costal margin distally darkened with brown scales. A distinct oblique brown stroke is present at the base of the cleft, not extending as far as the costa. The fringes inside the cleft include a mix of yellow and dark-grey sections. The second lobe has dark-brown filiform scales on the dorsum, with narrow white patches near the apex, and an apical part darkened with brown scales. The hindwings are grey, noticeably darker than the forewings, with a pale brown fringe. The hind legs are yellowish brown.

##### Description.

***Male genitalia*.** The valves are oval and asymmetric. The sacculus on the left valve is relatively narrow and slightly wider apically, and barely extends beyond the middle of the valve. The harpe on the same valve is slightly shorter than the sacculus, slightly bent, and narrows apically. The right sacculus has a plate extending from a bifurcate sclerotised arm. The uncus is straight, acute, and bent slightly along its length. The saccus is slightly moderately rounded. The anellus arms are asymmetric, with the left arm being slightly shorter. Both arms are apically acute. The aedeagus is thin, apically slightly bent, and 2/3 the length of the right valve.

***Female genitalia*.** The papillae anales are narrow and tapered. The posterior apophyses are thin. The distal margin of sternum VII is convex and rounded. The ostium is funnel-shaped, and the antrum is short, tubular, and shifted to the left. The ductus is as thick as the antrum, but membranous and relatively long. The bursa copulatrix is oval and lacks signa.

##### Differential diagnosis.

The male genitalia of the new species are similar to *Hellinsiaadumbratus* (Walsingham, 1881), but the latter differs in the absence of the tapered harpe on the right valve, having a narrower uncus, and an apically curved aedeagus. In the female genitalia, the convex sternum VII and the left-shifted antrum resemble *Hellinsiamadecasseus* (Bigot, 1964), but the new species differs by having a narrower ductus and lacking signa in the bursa copulatrix.

##### Distribution.

The species is only known from Cameroon.

##### Flight period.

The species was collected from February to May, and from October to December.

##### Etymology.

The species is named after Ekonjo, a village on the southern slope of Mount Cameroon, where it was collected. Several villagers contributed as field assistants and supported the project in various ways. This dedication aims to support the protection of Mount Cameroon unique biodiversity.

#### ﻿ Hellinsia mapanja

Taxon classificationAnimaliaLepidopteraPterophoridae

Ustjuzhanin & Kovtunovich sp. nov.

9CDD3324-B8CC-58A5-878E-81FF5224B70C

https://zoobank.org/AA56663D-6C2E-463F-B718-2D41DA942DAB

Figs 10–12

##### Type material examined.

***Holotype*** • ♂, (NECJU No. 241019), Cameroon, Crater Lake, 1,500 m a.s.l., Mount Cameroon, 4.1443°N, 9.0717°E, 21.XI.2016, V. Maicher, Sz. Sáfián, S. Janeček, R. Tropek leg.; ***Paratypes*** • 1♂, 1♀, and 3 other ex. Cameroon, Crater Lake, 23–29.IV.2017, V. Maicher, P. Potocký, S. Delabye leg.; 3♂, 1♀, 17–25.II.2017, P. Potocký, Sz. Sáfián, R. Tropek, J. Mertens, S. Janeček leg. • 3♂, Cameroon, Elephant camp, 17–22.II.2017, P. Potocký, Sz. Sáfián, R. Tropek, J. Mertens, S. Janeček leg.; 5 ex., 18–26.IV.2017, V. Maicher, P. Potocký, S. Delabye leg. • 2♂, 1♀, and 7 other ex., Cameroon, Mapanja, 10–15.V.2017, V. Maicher, P. Potocký, S. Delabye leg.; 2♂, 2♀, and 3 other ex., 23–28.X.2017, V. Maicher, S. Delabye leg. (CUK, NECJU).

**Figures 10–12.**
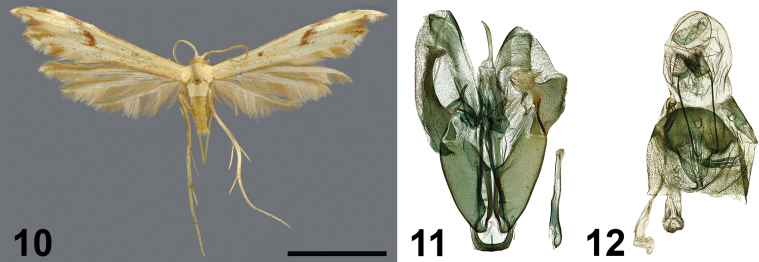
*Hellinsiamapanja* Ustjuzhanin & Kovtunovich, sp. nov. **10** adult male, holotype, NECJU**11** male genitalia, holotype, NEJCU, preparation slide no. 241019 **12** female genitalia, paratype. Scale bar: 5 mm.

##### External characters.

The wingspan ranges from 17 to 19 mm, with the holotype measuring 19 mm. The head is brown, while the thorax and tegulae are pale yellow. The labial palpi are brown, thin, and short, being half the length of the eye diameter. The antennae are yellow, interspersed with brown scales. The forewings are pale yellow, with an oblique elongated brown streak at the cleft base, reaching the costal margin of the first lobe. A more or less round brown discal spot is present near middle of the forewing. The costal margin of the forewing, up to the cleft, is mixed with tiny brown scales. A brown spot is situated medially along the costal margin on the first lobe. The fringe inside the cleft is yellow, with brown filiform scales distally. The second lobe is apically darkened with brown scales. The fringe along the outer edge of the forewing is pale yellow, except for a patch of pale brown filiform scales in its distal part under cleft. The hindwings are yellow and lack any pattern. The fringe on the hindwings is pale yellow, with alternating pale brown portions. The hind legs are pale yellow, interspersed with tiny brown scales.

##### Description.

***Male genitalia*.** The valves are asymmetric, with the left valve narrower and longer than the right valve. The saccular process on the left valve is well-developed and strongly sclerotised, extending far beyond the middle of the valve, and is widened and curved distally. The harpe is shaped like a short, slightly curved spike. The right valve is wide and slightly shorter than the left valve. The sacculus in the distal portion has a well-expressed sclerotized plate with processes on the sides. The uncus is narrow, slightly curved, and apically acute. The saccus is slightly rounded. The anellus arms are short and wide, with the left arm slightly shorter than the right. Both arms are apically narrow and acute. The aedeagus is thin, straight, and less than half as long as the right valve.

***Female genitalia*.** The papillae anales are short and triangular. The posterior apophyses are thin and long. The distal margin of sternum VII is convex and rounded, with a notch on the inner edge. The ostium is funnel-shaped, and the antrum is short, tubular, and shifted to the left. The ductus is short and membranous, smoothly transitioning into the narrow, elongated bursa copulatrix. Small, narrow, elongated signa are present laterally on the bursa.

##### Differential diagnosis.

Externally and in the genital structure, the species is similar to *Hellinsiaekonjo*. The wings are characterised by a pale yellowish tint and an oblique elongated brown streak that reaches the costal edge of the forewing, whereas in *H.ekonjo*, the wings are greyish brown and the elongated streak does not reach the costal edge of the forewing. In the male genitalia, the new species is distinguished by the valves of unequal length and width, the shape and size of the sacculus on the left valve, the shorter harpe, and the shorter, apically straight aedeagus. In the female genitalia, it is characterised by the signa in the bursa copulatrix and the small triangular papillae anales.

##### Distribution.

The species is only known from Cameroon.

##### Flight period.

The species was collected from February to May, and from October to November.

##### Etymology.

The species is named after Mapanja, a village on the southern slope of Mount Cameroon, where it was collected. Several villagers contributed as field assistants and supported the project in various ways. We strongly believe this dedication will also help protect the unique biodiversity of the region.

### ﻿Other species recorded on Mount Cameroon

#### ﻿*Agdistisclara Arenberger, 1986

*Agdistisclara* Arenberger, 1986: 189. (Type locality: Botswana).

**Material examined.** 2♂, 1♀ (CUK, NECJU), Cameroon, Ekonjo farmland, 19.II.2017, P. Potocký leg.

**Distribution.** Botswana, Namibia, Republic of South Africa, and Cameroon.

**Note.** New species for Cameroon.

#### ﻿*Deuterocopussocotranus Rebel, 1907

*Deuterocopussocotranus* Rebel, 1907: 115. (Type locality: Yemen, Socotra).

*Deuterocopusdeltoptilus* Meyrick, 1930: 565. (Type locality: Uganda).

*Deuterocopushenrioti* Bigot & Boireau, 2006: 16–17. (Type locality: Cote d’Ivoire).

**Material examined.** 1♀ (CUK), Cameroon, PlanteCam, 09–14.IV.2015, V. Maicher, Sz. Sáfián, S. Janeček, R. Tropek leg.

**Distribution.** Widely distributed species, besides several Afrotropical countries, it occurs also in Australasian, Oriental, and Palaearctic regions.

**Note.** New species for Cameroon.

#### ﻿*Titanoptilusprocerus Bigot, 1969

*Titanoptilusprocerus* Bigot, 1969: 182. (Type locality: West Kasai, Sankuru, Dimbelenge, Democratic Republic of Congo).

**Material examined.** 1♀ (CUK), Cameroon, PlanteCam, 09–14.IV.2015, V. Maicher, Sz. Sáfián, S. Janeček, R. Tropek leg.

**Distribution.** Congo and Cameroon.

**Note.** New species for Cameroon.

#### ﻿*Walsinghamiellaprolai (Gibeaux, 1994)

*Titanoptilusprolai* Gibeaux, 1994: 82. (Type locality: Comoros).

**Material examined.** 2♂ (CUK, NECJU), Cameroon, Bamboo Camp, 17–23.IV.2015, V. Maicher, Sz. Sáfián, S. Janeček, R. Tropek leg.

**Distribution.** Comoros, Republic of South Africa, Eswatini, Zimbabwe, Tanzania, and Cameroon.

**Note.** New species for Cameroon.

#### ﻿*Platyptiliabenitensis* Strand, 1913

*Platyptiliabenitensis* Strand, 1913: 64. (Type locality: Alén, Equatorial Guinea).

**Material examined.** 1♀, Cameroon, Crater Lake, 17–25.II.2017, P. Potocký, Sz. Sáfián, J. Mertens, Š. Janeček, R. Tropek leg.; 2♂, 1♀, 23–29.IV.2017, V. Maicher, P. Potocký, S. Delabye leg. • 1♀, Cameroon, PlanteCam, 09–14.IV.2015, V. Maicher, Sz. Sáfián, S. Janeček, R. Tropek leg. • 1♀, Cameroon, Mapanja, 25.X.2017, V. Maicher, S. Delabye leg. (CUK, NECJUK).

**Distribution.** Equatorial Guinea, Democratic Republic of Congo, Cote d’Ivoire, Cameroon, Kenya, Nigeria, Republic of South Africa, Sao Tome and Principe, Tanzania, and Uganda.

#### ﻿*Platyptiliadaemonica Meyrick, 1932

*Platyptiliadaemonica* Meyrick, 1932: 109. (Type locality: Jem-Jem Forest, Ethiopia).

**Material examined.** 1♂, Cameroon, Crater Lake, 24.XI.2016, V. Maicher, Sz. Sáfián, S. Janeček, R. Tropek leg.; 2♂, 17–25.II.2017, P. Potocký, Sz. Sáfián, R. Tropek, J. Mertens, S. Janeček leg.; 1♂, 23–29.IV.2017, V. Maicher, P. Potocký, S. Delabye leg. • 1♂, 1♀, Cameroon, Elephant Camp, 19–24.XI.2014; 3♂, 18–26.IV.2017, V. Maicher, P. Potocký, S. Delabye leg.; 2♂, 17–22.II.2017, P. Potocký, Sz. Sáfián, R. Tropek, J. Mertens, S. Janeček leg. • 3♂, Cameroon, Mann’s spring, 07–09.XI.2016, V. Maicher, Sz. Sáfián, S. Janeček, R. Tropek leg.; 1♂, 31.I.2017, P. Potocký, Sz. Sáfián, R. Tropek, J. Mertens, S. Janeček leg.; 1 ex., 13.VIII.2017, P. Potocký leg. • 2♂, Cameroon, Mapanja, 22.X.2017, V. Maicher, S. Delabye leg. (CUK, NECJU).

**Distribution.** Ethiopia, Rwanda, and Cameroon.

**Note.** New species for Cameroon.

#### ﻿*Platyptiliafletcheri Ustjuzhanin & Kovtunovich, 2016

*Platyptiliafletcheri* Ustjuzhanin & Kovtunovich, 2016: 2. (Type locality: Ruwenzori Range, Lake Mahoma, Uganda).

**Material examined.** 4♂, 1♀, Cameroon, Mann’s spring, 8–12.XI.2016, V. Maicher, Sz. Sáfián, S. Janeček, R. Tropek leg.; 4♂, 1♀, 31.I–04.II.2017, P. Potocký, Sz. Sáfián, R. Tropek, J. Mertens, S. Janeček leg. (CUK, NECJU).

**Distribution.** Uganda, Rwanda (Ustjuzhanin et al. 2016), and Cameroon.

**Note.** New species for Cameroon.

#### ﻿*Platyptiliagondarensis Gibeaux, 1994

*Platyptiliagondarensis* Gibeaux, 1994: 424. (Type locality: Gondar Province, Ethiopia).

**Material examined.** 1♀ (CUK), Cameroon, P&T antenna station, 28.III.2015, Sz. Sáfián leg.

**Distribution.** Ethiopia, Kenya, Uganda, and Cameroon.

**Note.** New species for Cameroon.

#### ﻿*Platyptiliamorophaea Meyrick, 1920

*Platyptiliamorophaea* Meyrick, 1920: 38. (Type locality: station 39, Kenya).

**Material examined.** 3♂, Cameroon, Mann’s spring, 08–11.XI.2016, V. Maicher, Sz. Sáfián, S. Janeček, R. Tropek leg.; 35 ex., 28.I–04.II.2017, P. Potocký, Sz. Sáfián, R. Tropek, J. Mertens, S. Janeček leg.; 3 ex., 16–21.IV.2017, V. Maicher, P. Potocký, S. Delabye leg. • 4 ex., Cameroon, Mapanja, 22–23.X.2017, V. Maicher, S. Delabye leg. (CUK, NECJU).

**Distribution.** Kenya, Burundi, Democratic Republic of Congo, Ethiopia, Malawi, Tanzania, and Cameroon.

**Note.** New species for Cameroon.

#### ﻿*Platyptiliamugesse Kovtunovich & Ustjuzhanin, 2014

*Platyptiliamugesse* Kovtunovich & Ustjuzhanin, 2014: 459. (Type locality: Mugesse Forest, Malawi).

**Material examined.** 2♀, Cameroon, Crater Lake, 17–25.II.2017, P. Potocký, Sz. Sáfián, R. Tropek, J. Mertens, S. Janeček leg. • 1♀, Cameroon, Mann’s spring, 31.I.2017 • 1♀, Cameroon, Mapanja, 23.X.2017, V. Maicher, S. Delabye leg. (CUK, NECJU).

**Distribution.** The species was found in Malawi (Kovtunovich et al. 2014) and Cameroon.

**Note.** New species for Cameroon.

#### ﻿*Platyptiliasciophaea Meyrick, 1920

*Platyptiliasciophaea* Meyrick, 1920: 40. (Type locality: Kenya).

**Material examined.** 1♀, Cameroon, Mann’s spring, 31.I.2017, P. Potocký, Sz. Sáfián, R. Tropek, J. Mertens, S. Janeček leg. (CUK, NEJCU).

**Distribution.** Kenya, Uganda, and Cameroon.

**Note.** New species for Cameroon.

#### ﻿*Platyptiliodesalbisignatula Strand, 1913

*Platyptiliodesalbisignatula* Strand, 1913: 65. (Type locality: Alén, Equatorial Guinea).

**Material examined.** 2♂, Cameroon, Bimbia-Bonadikombo, 27–28.XII.2014, V. Maicher, Sz. Sáfián, R. Tropek, S. Janecek, leg. • 1♂, Cameroon, Crater Lake, 17–25.II.2017, P. Potocký, Sz. Sáfián, R. Tropek, J. Mertens, S. Janeček leg. (CUK, NECJU).

**Distribution.** Equatorial Guinea and Cameroon.

**Note.** New species for Cameroon.

#### ﻿*Amblyptiliadireptalis (Walker, 1864)

*Oxyptilusdireptalis* Walker, 1864: 934. (Type locality: Cape Province, Republic of South Africa).

*Platyptiliaamblydectis* Meyrick, 1932: 108. (Type locality: Jem-Jem Forest, Ethiopia).

**Material examined.** 1♂, Cameroon, Crater Lake, 23–29.IV.2017, V. Maicher, P. Potocký, S. Delabye leg. 1♂, Cameroon, Elephant camp, 17–22.II.2017, P. Potocký, Sz. Sáfián, R. Tropek, J. Mertens, S. Janeček leg. • 2♂, Cameroon, Mann’s spring, 30.I.2017 • (CUK, NECJU).

**Distribution.** Republic of South Africa, Burundi, Democratic Republic of the Congo, Malawi, Tanzania, Zimbabwe, Kenya, Ethiopia; India, Sri Lanka, Cameroon, and China (Ustjuzhanin et al. 2021).

**Note.** New species for Cameroon.

#### ﻿*Stenoptilianatalensis Ustjuzhanin & Kovtunovich, 2010

*Stenoptilianatalensis* Ustjuzhanin & Kovtunovich, 2010: 695. (Type locality: Howick, KwaZulu Natal, Republic of South Africa).

**Material examined.** 1♂, Cameroon, Crater Lake, 02–17.II.2017, P. Potocký, Sz. Sáfián, R. Tropek, J. Mertens, S. Janeček leg. • 1♂, Cameroon, Mann’s spring, 12.XI.2016, V. Maicher, Sz. Sáfián, S. Janeček, R. Tropek leg.; 9 ex., 28–31.I.2017, P. Potocký, Sz. Sáfián, R. Tropek, J. Mertens, S. Janeček leg.; 1♂, 02.IV.2017, V. Maicher, P. Potocký, S. Delabye leg. • 1♂,1♀, Cameroon, 22–23.X.2017, V. Maicher, S. Delabye leg. • 1♂, 2♀, Cameroon, P&T antenna station, 28.III.2015, Sz. Sáfián leg. (CUK, NECJU).

**Distribution.** Republic of South Africa, Lesotho, Malawi, and Cameroon.

**Note.** New species for Cameroon.

#### ﻿*Vietteilusborbonica (Viette, 1957)

*Platyptiliaborbonica* Viette, 1957: 170. (Type locality: Reunion Island).

**Material examined.** 1♂,1♀, Cameroon, Crater Lake, 23–29.IV.2017, V. Maicher, P. Potocký, S. Delabye leg. • 1♂,1♀, Cameroon, Mann’s spring, 13–16.VIII. 2017, P. Potocký leg.; 2♀, Cameroon, Mapanja, 23–29.X.2017. V. Maicher, P. Potocký, S. Delabye leg.; 1♂, 11.XI.2016, V. Maicher, Sz. Sáfián, S. Janeček, R. Tropek leg. • 1♀, Cameroon, Mapanja, 14.V.2017, V. Maicher, P. Potocký, S. Delabye leg.; (CUK, NECJU).

**Distribution.** Reunion Island, Tanzania, and Cameroon.

**Note.** New species for Cameroon.

#### ﻿*Bipunctiphorusdimorpha (Fletcher, 1910)

*Platyptiliadimorpha* Fletcher, 1910: 401. (Type locality: Morne Blanc, Mahé, Seychelles).

*Platyptiliapatriarcha* Meyrick, 1912: 54. (Type locality: Mfongosi, Zululand, Republic of South Africa).

*Bipunctiphorusetiennei* Gibeaux, 1994: 57. (Type locality: above Cialos, Piton Bleu Forest, Réunion).

**Material examined.** 1♀, Cameroon, Crater Lake, 23–29.IV.2017, V. Maicher, P. Potocký, S. Delabye leg. • 1♂, 1♀, Cameroon, Mapanja, 11–12.V.2017, V. Maicher, P. Potocký, S. Delabye leg. (CUK, NECJU).

**Distribution.** Seychelles, Republic of South Africa, Reunion Island, Tanzania, Uganda, Zimbabwe, Kenya, Madagascar, Malawi, Sierra Leone, and Cameroon.

**Note.** New species for Cameroon.

#### ﻿*Stenoptilodestaprobanes (Felder & Rogenhofer, 1875)

*Amblyptiliataprobanes* Felder & Rogenhofer, 1875: plate 140, fig. 54. (Type locality: Ceylon [Sri Lanka]).

*Platyptilialegrandi* Bigot, 1962b: 86. (Type locality: Beau Vallon, Mahé, Seychelles).

*Stenoptilodesvittata* Service, 1966: 11. (Type locality: Nigeria).

**Material examined.** 1♂, Cameroon, Bamboo Camp, 17–23.IV.2015, V. Maicher, Sz. Sáfián, S. Janeček, R. Tropek leg. • 2♀, Cameroon, PlanteCam, 11–20.XII.2014, V. Maicher, Sz. Sáfián, S. Janeček, R. Tropek leg.; 4 ex., 29.I.–07.II.2016, Sz. Sáfián, R. Tropek, V. Maicher leg. (CUK, NECJU).

**Distribution.** Widespread throughout tropical and subtropical regions.

**Note.** New species for Cameroon.

#### ﻿*Sphenarchesanisodactylus* (Walker, 1864)

*Sphenarchesanisodactylus* Walker, 1864: 934. (Type locality: Ceylon [Sri Lanka]).

*Platyptiliapygmaeana* Strand, 1913: 64. (Type locality: Cameroon).

**Material examined.** 1♂, Cameroon, Crater Lake, 25.XI.2016, V. Maicher, Sz. Sáfián, S. Janeček, R. Tropek leg. • 1♀, Cameroon, Ekonjo, 21.X.2017, V. Maicher, S. Delabye leg. • 1♂, 2♀, Cameroon, PlanteCam, 11–18.XII.2014, V. Maicher, Sz. Sáfián, S. Janeček, R. Tropek leg.; 2 ex., 29.I.–07.II.2016, Sz. Sáfián, R. Tropek, V. Maicher leg. (CUK, NECJU).

**Distribution.** Widely distributed in tropical and subtropical regions.

#### ﻿*Procapperiainsomnis (Townsend, 1956)

*Capperiainsomnis* Townsend, 1956: 93. (Type locality: Nakuru, Kenya).

*Procapperiahackeri* Arenberger, 2002: 74. (Type locality: Yemen).

**Material examined.** 1♀ (CUK), Cameroon, PlanteCam, 11–18.XII.2014, V. Maicher, Sz. Sáfián, S. Janeček, R. Tropek leg.

**Distribution.** Kenya, Democratic Republic of the Congo, Yemen, Namibia, Zimbabwe, Republic of South Africa, Malawi, and Cameroon.

**Note.** New species for Cameroon.

#### ﻿*Megalorhipidaleucodactylus* (Fabricius, 1794)

*Pretophorusleucodactylus* Fabricius, 1794: 346. (Type locality: Virgin Islands).

*Pterophorusdefectalis* Walker, 1864: 943. (Type locality: Sierra Leone).

**Material examined.** 1♂ (CUK), Cameroon, Bamboo Camp, 14–23. II.2016, Sz. Sáfián leg.

**Distribution.** Widespread throughout tropical and subtropical regions.

#### ﻿*Inferuncusstrictiformis (Meyrick, 1932)

*Platyptiliastrictiformis* Meyrick, 1932: 251. (Type locality: Kampala, Uganda).

*Platyptiliaspiculivalva* Gielis, 1990: 120. (Type locality: E Usambara Mts, Amani, Tanzania).

**Material examined.** 1♂ (CUK), Cameroon, PlanteCam, 09–14.IV.2015, V. Maicher, Sz. Sáfián, S. Janeček, R. Tropek leg.

**Distribution.** Uganda, Tanzania, Congo, and Cameroon.

**Note.** New species for Cameroon.

#### ﻿*Inferuncustoxochorda (Meyrick, 1934)

*Platyptiliatoxochorda* Meyrick, 1934: 402. (Type locality: Sao Tome).

*Platyptiliapentheres* Bigot, 1969: 191. (Type locality: Equateur, Bokuma, Democratic Republic of Congo).

**Material examined.** 1♀ (CUK), Cameroon, Mann’s spring, 12.VIII.2017, P. Potocký, S. Janeček leg.

**Distribution.** Sao Tome and Principe, Congo, Democratic Republic of Congo, Tanzania, Ghana, Liberia, and Cameroon.

**Note.** New species for Cameroon.

#### ﻿*Exelastispumilio (Zeller, 1873)

*Mimeseoptiluspumilio* Zeller, 1873 (Type locality: Texas, Dallas, U.S.A.).

*Mimaeseoptilusgilvidorsis* Hedemann, 1896 (Type locality: Saint Croix, American Virgin Islands).

*Exelastisgilvidorsis* (Hedemann, 1896).

*Marasmarchaliophanes* Meyrick, 1886 (Type locality: Saint-Denis, Réunion).

*Exelastisliophanes* (Meyrick, 1886).

*Marasmarchatenax* Meyrick, 1913 (Type locality: Barberton, Mpumalanga, Republic of South Africa).

*Exelastistenax* (Meyrick, 1913).

*Exelastisbergeri* Bigot, 1969: 176. (Type locality: Democratic Republic of Congo).

**Material examined.** 1♂ (CUK), Cameroon, PlanteCam, 11–18.XII. 2014, V. Maicher, Sz. Sáfián, S. Janeček, R. Tropek leg.

**Distribution.** Democratic Republic of Congo, Republic of South Africa, Sierra Leone, Uganda, and Cameroon.

**Note.** New species for Cameroon. Ustjuzhanin et al. (2020b) mentioned *E.bergeri* and *E.tenax* as separate species. Nevertheless, these changes have never been formalised, and until a detailed revision, they remain synonyms of *E.pumilio*.

#### ﻿*Hellinsiaambo Ustjuzhanin & Kovtunovich, 2011

*Hellinsiaambo* Ustjuzhanin & Kovtunovich, 2011: 356. (Type locality: West Shewa, Ethiopia).

*Hellinsiaruhuruinia* Gielis, 2011: 51. (Type locality: Aberdare N.P., Ruhuruini Gates, Kenya).

**Material examined.** 1♂, Cameroon, Crater Lake, 17.II.2017, P. Potocký, Sz. Sáfián, R. Tropek, J. Mertens, S. Janeček leg. • 1♀, Cameroon, Ekonjo, 21.X.2017. V. Maicher, S. Delabye leg. • 1♂, Cameroon, Elephant Camp, 19–24.XI.2014, V. Maicher, Sz. Sáfián, S. Janeček, R. Tropek leg.; 1♂, 18–26.IV. 2017, V. Maicher, P. Potocký, S. Delabye leg.; 1 ex., 17–22.II.2017, P. Potocký, Sz. Sáfián, R. Tropek, J. Mertens, S. Janeček leg. • 1♂, Cameroon, Mann’s spring, 13.VIII.2017, P. Potocký leg.; 1♂, 30.I.2017, P. Potocký, Sz. Sáfián, S. Janeček, R. Tropek, leg.; 1♂, 15.VIII.2017, P. Potocký leg. • 1♂, 1♀, Cameroon, Mapanja, 22–23.X.2017, V. Maicher, S. Delabye leg. (CUK, NECJU).

**Distribution.** Ethiopia, Kenya, Rwanda, Uganda, Ghana, and Cameroon.

**Note.** New species for Cameroon.

#### ﻿*Picardiaeparches (Meyrick, 1931)

*Pterophoruseparches* Meyrick, 1931: 176. (Type locality: Butandiga, Uganda).

**Material examined.** 5 ex., Cameroon, Crater Lake, 17.II.2017, V. Maicher, Sz. Sáfián, S. Janeček, R. Tropek leg.; 10 ex., 23–29.IV.2017, V. Maicher, P. Potocký, S. Delabye leg. • 3 ex., Cameroon, Elephant camp, 18–26.IV.2017 • 3 ex., Cameroon, Mann’s spring, 16–21.IV.2016, leg.; 1 ex., 04.II.2017, P. Potocký, Sz. Sáfián, R. Tropek, J. Mertens, S. Janeček leg.; 1♀, 15.VIII.2017. P. Potocký leg. • 7 ex., Cameroon, Mapanja, 10–15.V.2017, V. Maicher, P. Potocký, S. Delabye leg.; 1♂, 23.X.2017, V. Maicher, S. Delabye leg. • 1♂, Cameroon, PlanteCam, 09–14.IV.2015, V. Maicher, Sz. Sáfián, S. Janeček, R. Tropek leg. (CUK, NECJU).

**Distribution.** Uganda, Kenya, Malawi, Zimbabwe, Tanzania (Kovtunovich et al. 2014), Zambia (Ustjuzhanin et.al. 2022), and Cameroon.

**Note.** New species for Cameroon.

#### ﻿*Picardiatropeki* Ustjuzhanin & Kovtunovich, 2022

**Material examined.** 1♂ (NECJU), Cameroon, PlanteCam, 09–14.IV.2015, V. Maicher, Sz. Sáfián, S. Janeček, R. Tropek leg.

**Distribution.** Cameroon.

**Note.** This species was previously described from the same material sampled on Mount Cameroon in a separate global overview of the genus *Picardia* (Ustjuzhanin et al. 2022).

#### ﻿*Pselnophorusbusoroensis Gielis, 2011

*Pselnophorusbusoroensis* Gielis, 2011: 51. (Type locality: Nyungwe NP, Busoro, Rwanda).

**Material examined.** 1♂, 1♀, Cameroon, Crater Lake, 23–29. IV. 2017, V. Maicher, P. Potocký, S. Delabye leg. • 1♂, 1♀, Cameroon, Elephant camp, 17–22.II.2017, P. Potocký, Sz. Sáfián, R. Tropek, J. Mertens, S. Janeček leg. • 1♀, Cameroon, Mann’s spring, 28.I.2017, P. Potocký, Sz. Sáfián, R. Tropek, J. Mertens, S. Janeček leg. (CUK, NECJU).

**Distribution.** Rwanda and Cameroon.

**Note.** New species for Cameroon.

#### ﻿*Adainamicrodactyla (Hübner, [1813])

*Alucitamicrodactyla* Hübner, [1813]: pl. 5, figs 26, 27. (Type locality: Europe).

*Oidaematophorusmadecasseus* Gibeaux, 1994: 130. (Type locality: Madagascar).

**Material examined.** 1♀ (CUK), Cameroon, Mapanja, 12.V.2017, V. Maicher, P. Potocký, S. Delabye leg.

**Distribution.** Madagascar, Democratic Republic of Congo, Tanzania, Malawi, and Cameroon, together with parts of Palaearctic and Palaeotropical regions, and New Guinea.

**Note.** New species for Cameroon.

#### ﻿*Pterophoruscleronoma (Meyrick, 1920)

*Alucitacleronoma* Meyrick, 1920: 41. (Type locality: Mt Kenya, Kenya).

**Material examined.** 1♀, Cameroon, Crater Lake, 24.XI.2016, V. Maicher, Sz. Sáfián, S. Janeček, R. Tropek leg.; 8 ex., 17–25.II.2017, P. Potocký, Sz. Sáfián, R. Tropek, J. Mertens, S. Janeček leg.; 1♂, 23–29.IV.2017, V. Maicher, P. Potocký, S. Delabye leg. • 2♂, Cameroon, Elephant camp, 17–22.II.2017, P. Potocký, Sz. Sáfián, R. Tropek, J. Mertens, S. Janeček leg.; 1♂, 18–26.IV.2017, V. Maicher, P. Potocký, S. Delabye leg. • 2♂, Cameroon, Mann’s spring, 8–12.XI.2016, P. Potocký, Sz. Sáfián, S. Janeček, R. Tropek leg. • 2♂, Cameroon, Mapanja, 22.X.2017, V. Maicher, S. Delabye leg. (CUK, NECJU).

**Distribution.** Kenya and Cameroon.

**Note.** New species for Cameroon.

#### ﻿*Cosmoclostisschouteni* Gielis, 1990

*Cosmoclostisschouteni* Gielis, 1990: 123. (Type locality: Daloa, Cote d’Ivoire).

*Pterophorusashanti* Arenberger, 1995: 250. (Type locality: Kumasi, Ghana).

**Material examined.** 1♂, 1♀ (CUK), Cameroon, Drink Gari, 06–15.II.2016, V. Maicher, Sz. Sáfián, R. Tropek leg.

**Distribution.** Cote d’Ivoire, Cameroon, Liberia, Nigeria, Ghana.

## ﻿Discussion

In this study, we report 34 species of Pterophoridae from Mount Cameroon, including four species new to science, and 25 species recorded in the country for the first time. These species also represent 14 genera not recorded in Cameroon previously: *Adaina*, *Agditis*, *Amblyptilia*, *Bipunctiphorus*, *Deuterocopus*, *Inferuncus*, *Platyptiloides*, *Procapperia*, *Pselnophorus*, *Stenoptilia*, *Stenoptilodes*, *Titanoptilus*, *Vietteilus*, and *Walsinghamiella*. These records highlight Mount Cameroon as a significant locality for plume moth diversity. Beyond the newly described species, the addition of numerous new records for Cameroon confirms the importance of Mount Cameroon as a previously overlooked site for studying Afrotropical moths (Maicher et al. 2016; Ustjuzhanin et al. 2018, 2020a, 2024; Delabye et al. 2020).

This study substantially increased the documented diversity of plume moths in this underexplored region, raising the number of Pterophoridae species known from Cameroon from 19 (De Prins and De Prins 2025) to 48. Consequently, Cameroon is now known to host more than 10% of the 429 known species from the Afrotropics (based on De Prins and De Prins 2025, plus the new species described here). This significant contribution emphasises the need for further surveys in understudied areas, such as Cameroon, to fully elucidate the diversity of Afrotropical Pterophoridae.

In addition to the new species descriptions, this study significantly expands the known distribution of several plume moth species. While some of the recorded species also occur in neighbouring countries, nine species (*P.daemonica*, *P.fletcheri*, *P.gondarensis*, *P.mugesse*, *P.sciophaea*, *V.borbonica*, *P.eparches*, *P.busoroensis*, and *P.cleronoma*) were previously known only from East Africa, *A.clara* was known only from South Africa, and two species (*W.prolai* and *S.natalensis*) were previously recorded from both of these distant regions. Although such scattered distributional patterns are documented in other moth groups (Maicher et al. 2016; Delabye et al. 2020) and better-studied taxa like butterflies (Larsen 2005), these range extensions more likely reflect the insufficient knowledge of plume moth distribution in Sub-Saharan Africa. Mount Cameroon is now the easternmost point in the known distribution for the West African species *C.schouteni*, and the westernmost point for the Central African species *T.procerus* and *P.albisignatula*, reflecting Cameroon’s position as a biogeographical crossroads between West and Central Africa. Nevertheless, for any biogeographical conclusions and interpretations, much better faunistic and taxonomic knowledge of the Pterophoridae Afrotropical diversity is needed.

Mount Cameroon continues to demonstrate its status as a critical biodiversity hotspot within the Afrotropics. Although the diversity at the site is not as exceptional for Pterophoridae as for Alucitidae (Ustjuzhanin et al. 2018, 2020a, 2024), the number of endemic plume moth species and the biogeographically significant records align with patterns seen in butterflies (Larsen 2005), other moths (Yakovlev and Sáfián 2016), and some other non-lepidopteran taxa (Cable and Cheek 1998; Damaška et al. 2022; Hlaváč et al. 2023; Janeček and Janečková 2025). This can be attributed to the mountain’s wide range of habitats, from littoral forests and lowland rainforests to montane cloud forests and subalpine grasslands, with large areas under minimal anthropogenic pressure. Its position at the border of two biogeographic regions, along with its isolation from other montane ranges, further contributes to the area’s high level of endemism.

Our findings confirm the critical need to protect the ecosystems of Mount Cameroon. While parts of the region remain relatively well-preserved inside Mount Cameroon National Park, ongoing threats from habitat degradation and deforestation persist, particularly in lowland areas (Cable and Cheek 1998; Ferenc et al. 2018; Dongmo et al. 2023) and on the more densely populated eastern and southeastern slopes, where the regional capital, Buea, is located (Cable and Cheek 1998). Additionally, climate change poses significant risks to high-elevation species uniquely adapted to cooler, more stable environments (e.g., Ivory et al. 2016; Linck et al. 2021). Continued conservation efforts, particularly in the form of habitat protection within the national park and sustainable land-use practices in its buffer zones, will be key to preserving the biodiversity of this globally important site. We hope that dedicating some of the newly described species to local communities will foster greater appreciation for the biodiversity of their ecosystems. Our findings reinforce the urgent need to prioritise the conservation of the Mount Cameroon region to ensure the long-term survival of its unique ecosystems and the species they harbour.

## Supplementary Material

XML Treatment for Titanoptilus bigoti

XML Treatment for Titanoptilus murkwe

XML Treatment for Hellinsia ekonjo

XML Treatment for Hellinsia mapanja

## ﻿Additional information

### Author ORCIDs

Peter Ustjuzhanin https://orcid.org/0000-0002-5222-2241

Vasily N. Kovtunovich https://orcid.org/0000-0001-5091-4263

Sylvain Delabye https://orcid.org/0000-0003-0911-9721

Vincent Maicher https://orcid.org/0000-0002-9147-3529

Szabolcs Sáfián https://orcid.org/0000-0002-0614-4203

Eric B. Fokam https://orcid.org/0000-0003-4531-3580

Robert Tropek https://orcid.org/0000-0001-7499-6259

### Data availability

All of the data that support the findings of this study are available in the main text.

## ﻿Appendix 1

**List of all species of Pterophoridae recorded in Cameroon**

The list follows the AfroMoths online database (de Prins and de Prins 2025), with the addition of the species included in this paper (new country records are marked by an asterisk).

**Adainamicrodactyla* (Hübner, [1813])

**Agdistisclara* Arenberger, 1986

**Amblyptiliadireptalis* (Walker, 1864)

*Antarchestessmanni* (Strand, 1913)

**Bipunctiphorusdimorpha* (Fletcher, 1910)

*Cosmoclostisschouteni* Gielis, 1990

**Deuterocopussocotranus* Rebel, 1907

*Exelastiscrudipennis* (Meyrick, 1932)

**Exelastispumilio* (Meyrick, 1913)

*Fletcherellaniphadarcha* (Meyrick, 1930)

**Hellinsiaambo* Ustjuzhanin & Kovtunovich, 2011

**Hellinsiaekonjo* Ustjuzhanin & Kovtunovich, sp. nov.

*Hellinsialienigianus* (Zeller, 1852)

**Hellinsiamapanja* Ustjuzhanin & Kovtunovich sp. nov.

**Inferuncusstrictiformis* (Meyrick, 1932)

**Inferuncustoxochorda* (Meyrick, 1934)

*Marasmarchasisyrodes* Meyrick, 1921

*Megalorhipidaleucodactylus* (Fabricius, 1794)

**Picardiaeparches* (Meyrick, 1931)

*Picardiatropeki* Ustjuzhanin & Kovtunovich, 2022

*Platyptiliabenitensis* Strand, 1913

**Platyptiliadaemonica* Meyrick, 1932

*Platyptiliafarfarellus* (Zeller, 1867)

**Platyptiliafletcheri* Ustjuzhanin & Kovtunovich, 2016

**Platyptiliagondarensis* Gibeaux, 1994

**Platyptiliamorophaea* Meyrick, 1920

**Platyptiliamugesse* Kovtunovich & Ustjuzhanin, 2014

**Platyptiliasciophaea* Meyrick, 1920

**Platyptiliodesalbisignatula* Strand, 1913

**Procapperiainsomnis* (Townsend, 1956)

**Pselnophorusbusoroensis* Gielis, 2011

*Pterophorusalbidus* (Zeller, 1852)

*Pterophoruscandidalis* (Walker, 1864)

**Pterophoruscleronoma* (Meyrick, 1920)

*Pterophorusceraunia* (Bigot, 1969)

*Pterophoruslampra* (Bigot, 1969)

*Pterophorusrhyparias* (Meyrick, 1908)

*Pterophorusspissa* (Bigot, 1969)

*Pterophorusvirgo* (Strand, 1913)

*Sphenarchesanisodactylus* (Walker, 1864)

*Stenodacmawahlbergi* (Zeller, 1852)

**Stenoptilianatalensis* Ustjuzhanin & Kovtunovich, 2010

**Stenoptilodestaprobanes* (Felder & Rogenhofer, 1875)

**Titanoptilusbigoti* Ustjuzhanin & Kovtunovich, sp. nov.

**Titanoptilusmurkwe* Ustjuzhanin & Kovtunovich, sp. nov.

**Titanoptilusprocerus* Bigot, 1969

**Vietteilusborbonica* (Viette, 1957)

**Walsinghamiellaprolai* (Gibeaux, 1994)
